# Comparison of Optical and Power Doppler Ultrasound Imaging for Non-Invasive Evaluation of Arsenic Trioxide as a Vascular Disrupting Agent in Tumors

**DOI:** 10.1371/journal.pone.0046106

**Published:** 2012-09-28

**Authors:** Mustafa K. Alhasan, Li Liu, Matthew A. Lewis, Jennifer Magnusson, Ralph P. Mason

**Affiliations:** Department of Radiology, University of Texas Southwestern Medical Center, Dallas, Texas, United States of America; Okayama University, Japan

## Abstract

Small animal imaging provides diverse methods for evaluating tumor growth and acute response to therapy. This study compared the utility of non-invasive optical and ultrasound imaging to monitor growth of three diverse human tumor xenografts (brain U87-luc-mCherry, mammary MCF7-luc-mCherry, and prostate PC3-luc) growing in nude mice. Bioluminescence imaging (BLI), fluorescence imaging (FLI), and Power Doppler ultrasound (PD US) were then applied to examine acute vascular disruption following administration of arsenic trioxide (ATO).

During initial tumor growth, strong correlations were found between manual caliper measured tumor volume and FLI intensity, BLI intensity following luciferin injection, and traditional B-mode US. Administration of ATO to established U87 tumors caused significant vascular shutdown within 2 hrs at all doses in the range 5 to 10 mg/kg in a dose dependant manner, as revealed by depressed bioluminescent light emission. At lower doses substantial recovery was seen within 4 hrs. At 8 mg/kg there was >85% reduction in tumor vascular perfusion, which remained depressed after 6 hrs, but showed some recovery after 24 hrs. Similar response was observed in MCF7 and PC3 tumors. Dynamic BLI and PD US each showed similar duration and percent reductions in tumor blood flow, but FLI showed no significant changes during the first 24 hrs.

The results provide further evidence for comparable utility of optical and ultrasound imaging for monitoring tumor growth, More specifically, they confirm the utility of BLI and ultrasound imaging as facile assays of the vascular disruption in solid tumors based on ATO as a model agent.

## Introduction

Vascular disruption has been proposed as a therapy for solid tumors based on the principle of starving cancer of a supply of nutrients [1], [2], [3]. Indeed, many pre-clinical studies have reported successful application of antivascular agents particularly in combination with other therapies [4], [5], [6], [7], [8], [9]. However, to date clinical trials have been less successful and new agents are actively sought [10]. Non-invasive imaging techniques promise insight into vascular patency and efficient screening of dose response and therapeutic combinations. Recent development of small animal optical imaging including bioluminescence (BLI) and fluorescence (FLI), and high frequency ultrasound (US) imaging including power Doppler (PD) allows relatively cheap, easy and efficient observation of tumor growth and vasculature [2], [11], [12], [13], [14].

Optical imaging is particularly easy to implement and both BLI and FLI have become routine in cancer research notably for examining tumor growth, spread and response to therapy [11], [13], [15]. We recently demonstrated that dynamic BLI could be used to examine the effect of anti-vascular agents such as Combretastatin A4 phosphate on solid tumors in mice and demonstrated correlations with more traditional MRI, immunohistochemical and histological findings [16]. By analogy with dynamic contrast enhanced (DCE) MRI or CT, vascular disruption inhibits the ability of luciferin substrate to reach tumor cells yielding diminished bioluminescence.

While optical imaging is valuable in the pre-clinical setting, application to patients is less feasible and in this case ultrasound imaging offers relatively cost-effective measurement of both anatomy and vascular perfusion. New pre-clinical instruments offer high frequency transducers and 40 MHz Power Doppler ultrasound allows non-invasive detection of blood flow changes following a treatment. The use of PD to detect such changes *in vivo* with respect to anti-vascular agents has been reported previously [14], [17], [18] and is compared with optical imaging here.

In this study, arsenic trioxide (ATO) was used as a model vascular disrupting agent (VDA) on three different human tumor types including brain, breast, and prostate cell lines and assessed using BLI, FLI, and PD US. We investigated correlations between BLI and FLI signal intensities in dual reporter gene transfected cells and explored the correlation between BLI and Power Doppler ultrasound to reveal the acute effects of ATO on tumor vasculature.

## Materials and Methods

### Cell culture

U87 MG (human brain tumor, ATCC, Rockville, MD) and MCF7 (human breast tumor, ATCC) cell lines were transfected sequentially to stably express luciferase and mCherry genes and cultured using DMEM medium. PC3 (human prostate tumor, ATCC) cells were transfected with luciferase gene alone and cultured using HAM'S F12 medium according to ATCC cell culture protocol. Both media were supplemented with 10% FBS, 1% *L*-glutamine and 1% penicillin-streptomycin solution.

### Arsenic Trioxide preparation

A stock solution of 1% ATO was prepared by dissolving As_2_O_3_ (99.995% trace metals basis- 202673-5G; Sigma-Aldrich, St. Louis, MO) in distilled water with continuous stirring for five days and gentle heat for 2 hrs on the last day and then stored at 4°C. A second stock solution was prepared with 5% dextrose in 0.9% NaCl and used to dilute the ATO solution to the desired concentration. Dextrose was added to reduce the toxicity of ATO, as suggested [19].

### Tumor implantation and drug administration

All procedures were approved by the UT Southwestern Institutional Animal Care and Use Committee. Twenty one athymic nude mice (12 male and 9 female, 5–6 weeks old) were obtained from National Cancer Institute (NCI, Frederick, MD) and housed in a specific pathogen free facility. Tumors were generated by implanting cells of the three tumor cell lines U87-luc-mCherry, MCF7-luc-mCherry, and PC3-luc subcutaneously. U87 cells (1×10^6^ cells/mouse) were implanted on the back of the mice, while MCF7 and PC3 (2×10^6^ cells/mouse for each line) were implanted in the right flank. Caliper measurements were applied using the ellipsoid volume equation (height×width×length×π/6) to estimate tumor volume. Six MCF7-Luc-mCherry mice were assigned for histology. Various doses of ATO in the range 5 to 10 mg/kg or saline were administrated IP.

### Imaging protocols

BLI and FLI were performed using IVIS® Spectrum or Lumina II instruments (Caliper Life Sciences, Hopkinton, MA). On each occasion FLI was performed before BLI to avoid possible interference. Optimal detection of mCherry was achieved with λ_ex_ = 570 nm and λ_em_ = 620 nm with f-stop 1, pixel binning 8 and 0.5 s exposure time.

For BLI, sodium *D*-luciferin (80 µL, 40 mg/ml, sodium salt, Gold Biotechnology, St Louis, MO) was injected subcutaneously in the fore-back neck region, as described previously [20]. During tumor growth, a single image was acquired 10 minutes after substrate injection. On the day of treatment, images were acquired before saline or ATO treatment, and 2 hrs, 4 hrs, 6 hrs and 24 hrs afterwards. In this case, dynamic BLI was performed over a period of 16 to 30 minutes to observe evolution of signal intensity. The same mice were observed for control saline and ATO tests with the 24 hr saline time point corresponding to baseline ATO. Fresh luciferin was administered at each time point. BLI was applied to all three tumor types and additionally FLI for the U87-luc-mCherry and MCF7-luc-mCherry tumors. US imaging was performed after optical imaging for selected PC3-luc and MCF7-luc-mCherry tumors.

Ultrasound imaging was performed using a VisualSonics Vevo 770 High-Resolution Imaging System (Visual Sonics Inc, Toronto, Ontario, Canada) with a 40 MHz probe in B mode to identify the tumor region. Power Doppler mode was additionally performed at various time points (pre, 2 hrs, 4 hrs, 6 hrs, 24 hrs after drug or saline) to quantify the blood flow in each single slice of the tumor to provide a volumetric quantification.

### Data analysis

For BLI and FLI regions of interest (ROI) were chosen for each tumor. Total photon flux (φ = photon count/(image acquisition time x area)) was calculated for each mouse using the optical instrumentation software (Living Image 4.2). Signal intensities obtained for control and treated mice with different doses were compared before and after treatment. For PD US an ROI was selected in each single slice using the Vevo 770 V3.0.0 software and 3D reconstruction was performed to measure the tumor size. The percent of vascularity (PV) for power Doppler mode was calculated for the entire tumor volume using the ultrasound manufacturer's software.

### Statistical Analysis

Student's t-tests were used for comparisons between signal intensities for BLI and FLI, and PV values for PD ultrasound before, and after treatment. P<0.05 was considered significant. The square of the correlation coefficient (*R* ^2^) was determined for testing correlations between imaging modalities. Data following treatment were normalized to the baseline values (pre-treatment).

### Immunohistochemistry

CD31 staining: after imaging at each time point, the blue fluorescent dye Hoechst 33342 (10 mg/kg, Molecular Probes, Eugene, OR, USA) was injected into the tail vein of selected anesthetized mice, and the tumors were excised 1 min later. Tumor tissue was frozen in OCT (Sakura Finetek, Torrance, CA, USA) and then stored at −80°C. Cryosections (8 µm) were cut and fixed with 4% paraformaldehyde for 15 mins at room temperature. The tissue was then washed three times in PBS for 5 minutes. After blocking with normal goat serum for 3 hours, the slides were incubated with primary rat anti-mouse CD31 antibody (1∶1000, BD Pharmingen, USA) overnight at 4°C. Slides were rinsed three times at 5-minute intervals with PBS and incubated with Alexa Fluor 488 goat anti-rat antibody (1∶1000, Molecular Probes, Eugene, OR) for two hours in the dark. The slides were washed 5 times using PBS for 5 minutes each cycle. The slides were prepared with fluorescent mounting medium (Dako North America, Carpentaria, CA, USA), and imaged using an LSM 510 Meta confocal microscope (Carl Zeiss Microscopy, Germany). Overlay analysis of the CD31 antibody with Hoechst 33342 was performed using ImageJ (NIH).

Caspase-3 activity: additional sections were blocked with normal donkey serum for 3 hours, and slides incubated with cleaved caspase-3 (Asp175) primary antibody (1∶4000, Cell Signaling Technology, Beverly, MA, USA) overnight at 4°C. Slides were rinsed three times at 5 minutes each with PBS and incubated with Alexa Fluor 488 donkey anti-rabbit antibody (1∶1000, Molecular Probes, Eugene, OR, USA) for two hours in the dark. Further preparation was performed as for CD31 above. Photomicrography was achieved using a Leica DM2000 photomicroscope equipped with brightfield epi-fluorescence, incident angle darkfield illumination and an Optronics Microfire digital CCD color camera interfaced with Macintosh G4 computer. Images were captured using PictureFrame 2.0 acquisition and software (Optronics, Inc. Goleta, CA, USA).

H&E: Whole mount serial sections were cut at 8 µm and microscopy performed with the Leica DM2000. Imaging used a Microtek ArtixScann 400tf film scanner with PathScan Enabler slide holder (Meyer Instruments, Houston, TX, USA).

## Results

### Tumor growth curves

**Figure 1:**
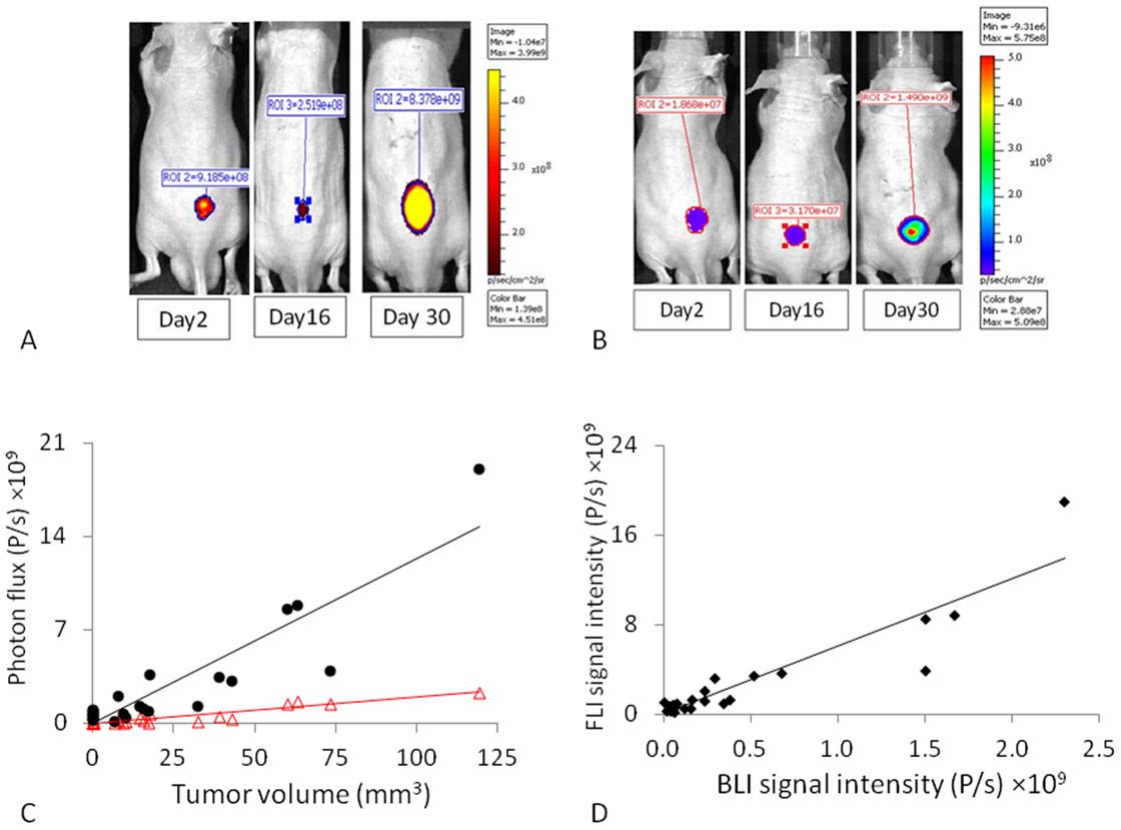
Optical detection of U87-mCherry-luc tumor growth. A) FLI (λ_Ex_ = 570 nm and λ_Em_ = 620 nm) showed tumor growth on the back of a nude mouse. B) Corresponding BLI acquired 10 mins after administration of sodium *D*-luciferin (80 µL, 40 mg/ml). C) Correlation between signal intensities and caliper measured tumor volume for a group of six U87-mCherry-Luc tumors; FLI (•; R^2^>0.82) and BLI (Δ; R^2^>0.86); D) Correlation between FLI and BLI photon signal intensities at each measurement time (R^2^>0.86).

Six mice implanted with U87-luc-mCherry cells were imaged using FLI and BLI over a period of 30 days (Figure 1 A, B). The maximum fluorescence for mCherry was observed using λ_ex_ = 570 nm and λ_em_ = 620 nm. Fluorescent signal intensity initially decreased over about two weeks following implantation when the tumors were subpalpable and then increased exponentially (Figure S1A). Fluorescence correlated strongly with caliper measured tumor volume (R^2^>0.82, Figure 1C; Table S1). Following administration of luciferin SC in the neck region of the mice, BLI signal increased over a period of about 20 mins. As for FLI, the BLI signal decreased and remained low during the first two weeks following implantation and then increased (Figures 1B & S1B). A strong correlation was determined between light emission and caliper measured tumor volume (R^2^>0.86; Figure 1C). As a corollary a strong correlation was found between FLI and BLI on each occasion (R^2^>0.84; Figure 1D, Table S1). Repeat imaging following saline injection IP showed highly reproducible signal intensities for both FLI and BLI (Figure S1C, D). Residual BLI signal was found to be <1% 2 hrs after luciferin administration, and therefore insignificant in terms of successive BLI signals or FLI measurements. Similar correlations were found for MCF7-luc-mCherry (n = 3) and PC3-luc (n = 3) (Figure S2, Table S1).

### Dose response in U87-luc-mCherry human brain tumor xenografts

**Figure 2:**
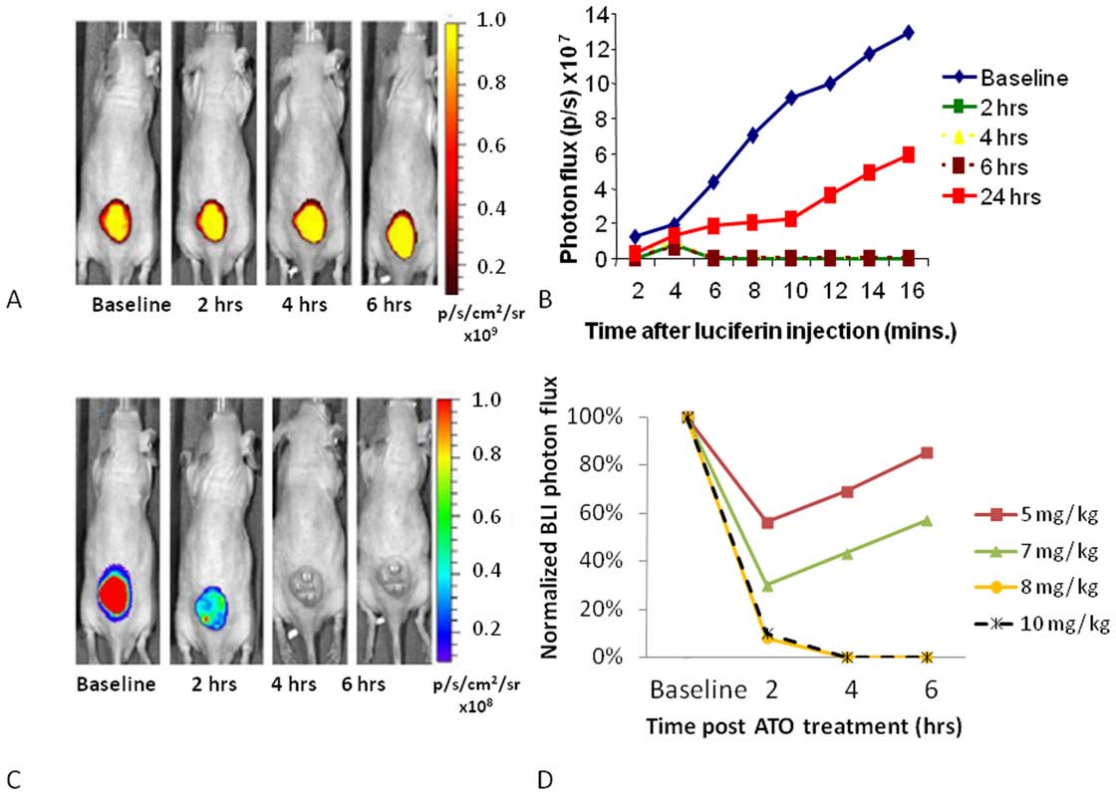
Optical assessment of vascular disruption in U87-mCherry-luc tumors. Optical imaging was performed at various times before and after administration of ATO. On each occasion FLI was performed first and then *D*-luciferin was administered SC in the neck and BLI was performed over a period of 16 mins. **A**) Sequential FLI with respect to a dose of 8 mg/kg ATO administered IP, **B**) Corresponding time course of bioluminescent signal evolution in this mouse following luciferin injection at respective times, **C**) BLI signal intensity at the 10 minute time point following luciferin administration, **D**) Normalized BLI signal intensity at 10 minute time point after administration of luciferin indicating vascular shutdown following various doses of ATO.

Following the control study with saline the same mice were used to evaluate response to ATO. Mice bearing U87-luc-mCherry tumors were injected IP with doses of ATO ranging from 5 to 10 mg/kg and sequential FLI and BLI acquired up to 24 hrs (Figure 2). FLI showed essentiality no change in the signal intensity up to 6 hrs even at the highest doses (Figure 2A). Following 8 mg/kg BLI showed significantly lower light emission, though with some recovery after 24 hrs (Figure 2 B, C). Both 5 and 7 mg/kg showed a reduction in the BLI signal intensity at 2 hrs, but with recovery by 4 and 6 hrs (Figure 2D). Both 8 and 10 mg/kg showed greater effect with signal remaining depressed at 24 hrs post treatment (Figure 2D). However, 10 mg/kg was unacceptably toxic with one mouse dying within 6 hrs. Relative BLI signal intensity for groups of mice is shown in Figure 2D, where each tumor was normalized to its baseline signal intensity and to the control value, which was considered to remain constant. Having established an effective dose for acute vascular shutdown, the methods were applied to additional tumor types and Power-Doppler Ultrasound added.

### Comparison of BLI, FLI and PD-US

MCF7-luc-mCherry showed similar behavior to U87 in terms of FLI and BLI signal versus tumor growth (Figure S2; Table S1). High frequency ultrasound showed effective contrast revealing the tumors and allowing estimates of tumor volume (Figure S3). A strong correlation was found between ultrasound and caliper estimates of tumor volume (R^2^>0.8).

**Figure 3:**
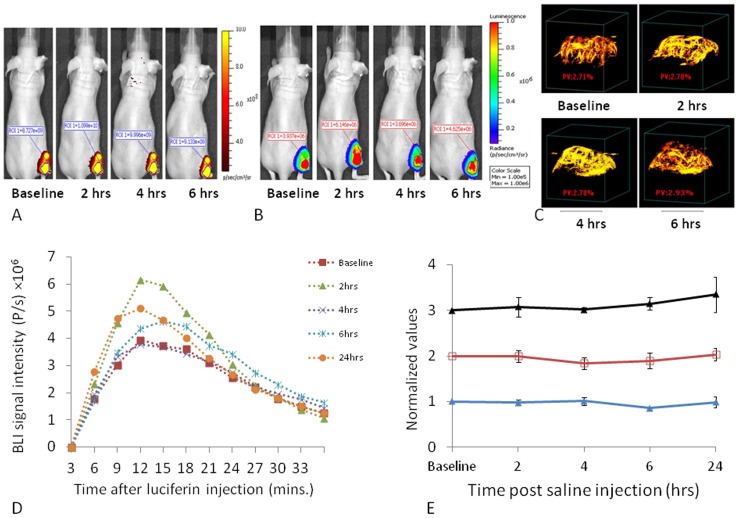
Reproducibility of sequential images in MCF7-mCherry-luc tumors. A group of nine MCF7-mCherry-luc tumors was repeatedly observed by FLI, BLI, and PD US following injection of 100 µl saline IP. A) Sequential FLI for a representative mouse, B) BLI from the same mouse showing images acquired 10 mins after administration of fresh luciferin on each occasion, c) PD US showing MIP (maximum intensity projection) observed at 40 MHz, D) Variation in dynamic bioluminescent signal intensity from the same tumor as in A,B and C, on five sequential occasions over 24 hrs, E) Comparison of signal stability based on FLI (red **□**), BLI (blue ▴) and PD-US (black ▴) normalized values are presented mean ± SE for the whole group.

With respect to saline injection highly reproducible BLI, FLI and PD US images were observed over a period of 24 hrs following injection of saline IP (Figure 3). Following luciferin injection BLI increased over the first 10 mins and then tended to decline over the following 20 minutes, having peaked somewhat more rapidly than for U87 tumors (*c.f.* Figures 2B and 3D). Curves were quite similar at each time point post saline injection following administration of fresh luciferin on each occasion with no significant differences (P>0.05; Figure 3E). FLI and PD-US also showed highly reproducible data with no signal changes over 24 hrs (Figure 3E).

**Figure 4:**
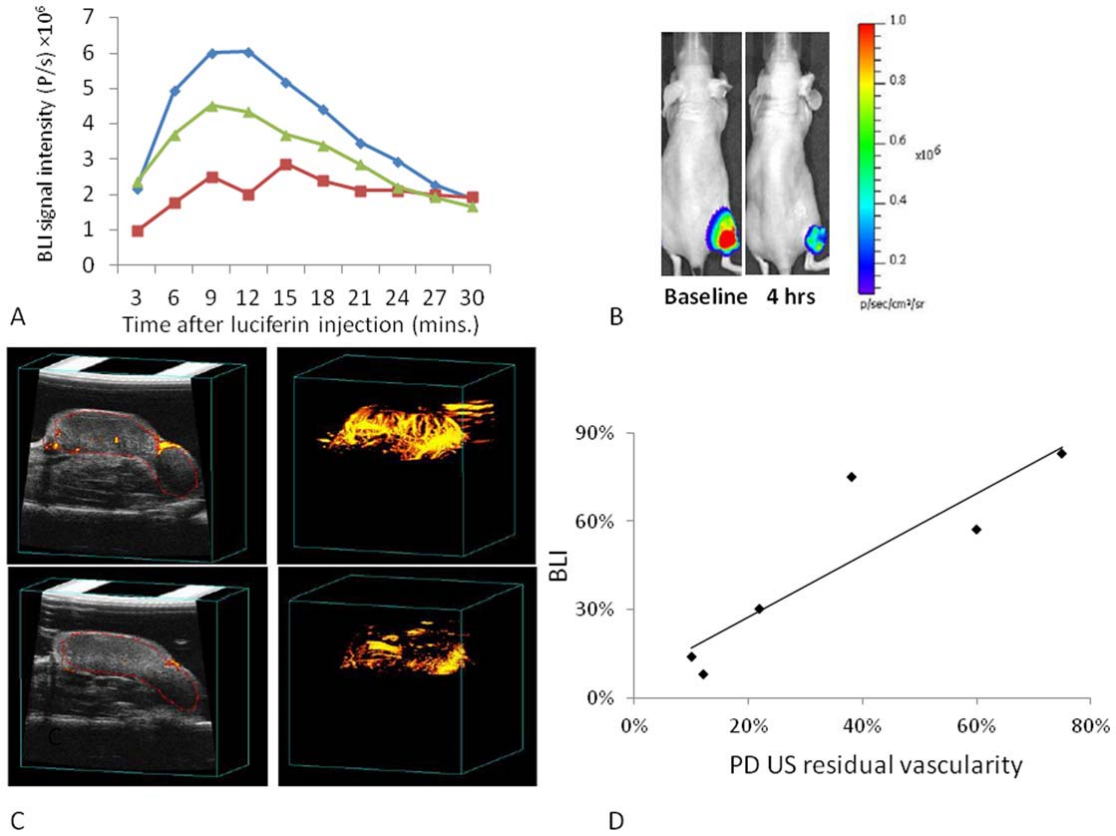
Vascular disruption assessed by BLI and PD-US in MCF7-mCherry-luc tumors with respect to ATO (8 mg/kg). A) Variation in bioluminescent signal intensity from a representative tumor on sequential occasions over 24 hrs: blue ♦ baseline; red ▪ 4 hrs; green ▴ 24 hrs. B) BLI acquired 10 mins after administration of luciferin. C) PD US images are presented as single slice (left) and PD maximum intensity projection (right) before and 4 hrs after treatment with ATO. D) Comparison of PD US and BLI in MCF7-mCherry-luc tumors as fractional signal versus baseline.

**Figure 5:**
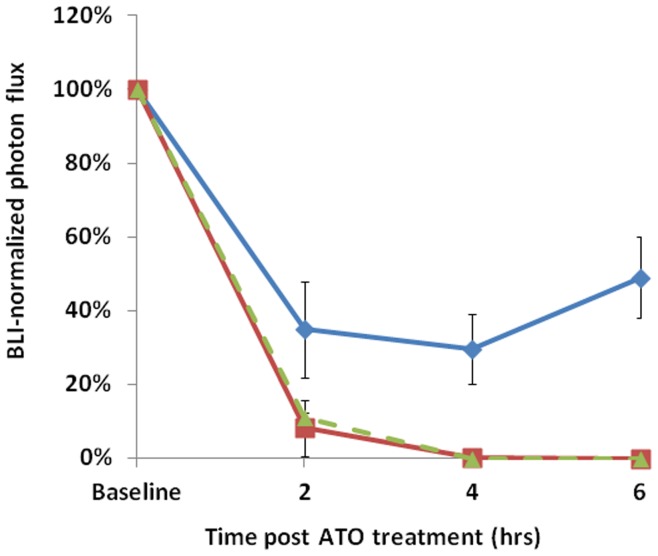
Comparative vascular shutdown following ATO (8 mg/kg) to mice bearing various tumors. Relative BLI signal intensity observed for tumors 10 mins after administration of luciferin with respect to drug treatment in three different types: blue ♦ MCF7-mCherry-luc; green ▴U87-mCherry-luc; red ▪ PC3-luc; mean values ± SE.

Repeat imaging every 2 hrs with any of the modalities was highly successful in the control state. Past experience has shown that repeated long term anesthesia with respect to vascular disrupting agents could cause animal death and therefore separate mice were imaged 2, 4, and 6 hrs after administration of ATO with each tumor serving as its own control. At each time point following administration of ATO, light emission was significantly reduced, as shown for a representative mouse in Figure 4, which showed a 65% decrease in peak light emission 4 hrs after ATO at 8 mg/kg. Ultrasound showed a similar reduction in the percent of vascularity (Figure 4C). Since both BLI and Power Doppler ultrasound are non-invasive, baseline and response values could be compared for each techniques and a strong correlation was found (R^2^>0.77) following ATO (8 mg/kg; Figure 4D). The effect of ATO (8 mg/kg) on the BLI signal intensity was found to be significant (P<0.05) 2–6 hrs after the treatment for all cell lines (Figure 5), but there was substantial recovery by 24 hrs and differences were no longer significant from baseline (P>0.05). For MCF7-luc-mCherry tumors the largest effect was seen after 4 hrs with recovery starting by 6 hrs post treatment. Significantly diminished BLI signal was also seen in the PC3-luc tumor following ATO (Figure S4). Since PD US was applied to only two PC3 tumors, the data were combined with the MCF7 results for comparison with BLI (Table S1)

**Figure 6:**
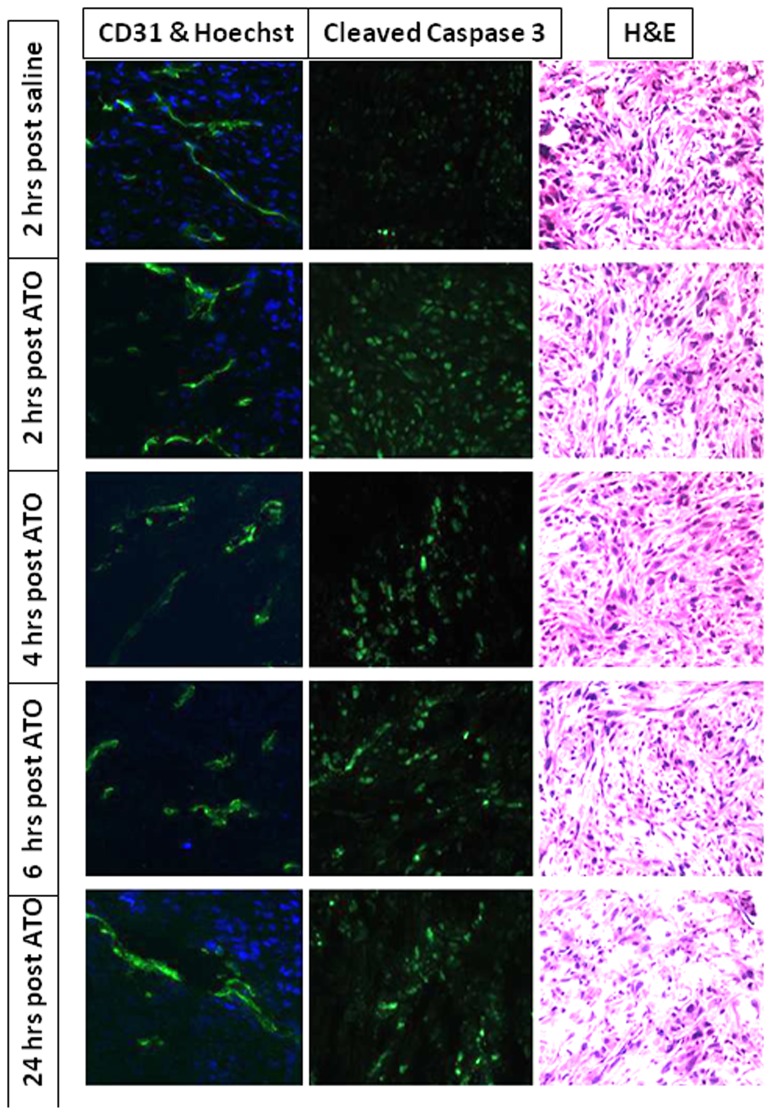
Histology showing vascular impairment and apoptosis in MCF7-mCherry-luc tumors. Sections were obtained from a series of mice sacrificed at various times after ATO (8 mg/kg). Hoechst stain shows reduced perfusion 2–6 hrs following ATO and H&E stain shows increased necrosis after 24 hrs. Left column: vascular extent (CD31; green) and perfusion (Hoechst 33342; blue). Middle column: Caspase-3 activity indicating apoptosis. Right column: H&E.

Histology confirmed that perfusion was compromised 2 to 6 hrs post ATO. ATO is reported to cause both vascular effects and induce apoptosis, as confirmed by IHC in a group of five MCF7-luc-mCherry tumors, which were excised at various times after ATO (Figure 6). Vascular density (estimated by anti-CD31 staining) was quite constant at all time points, but perfusion (based on Hoechst dye distribution) was clearly much lower 4 to 6 hrs after ATO (8 mg/kg). By 24 hrs there was evidence for reperfusion. Caspase 3 activity indicating apoptosis was somewhat elevated at all times following ATO and there was clear evidence for necrosis by 24 hrs.

## Discussion

Acute vascular response was observed in all three tumor types within two hours of administration of ATO IP. Non-invasive BLI and PD US each revealed vascular shutdown with considerable recovery 24 hr later. The effect was dose dependant in terms of fractional reduction in vascular perfusion and recovery.

All three imaging techniques (BLI, FLI, and US) accurately reported tumor growth by comparison with caliper measurements. The optical approaches have the advantage of revealing even sub-palpable volumes, though they do require that cells be transfected to express reporter genes. Tight correlation was observed between each of the techniques for measurements over a period of 30 days and volumes up to 200 mm^3^ (Figures 1, S2, S3). Measurements were highly reproducible in individual tumors as shown for repeat measurements following saline injection IP (Figure 3). Correlation between FLI and BLI has been reported previously by others, but those studies used cell lines separately transfected to express luciferase of fluorescent protein, as opposed to the double transfection examine here. Notably, Choy *et al.* [21] compared MC38-GFP and MC38-luc murine colon adenocarcinoma and reported close correlation between the methods and caliper measured tumor volume. Caceres *et al.* [22] compared MCF7 cells transfected GFP or luciferase and found close correlation between BLI and FLI, but noted that BLI was superior for evaluating metastatic spread and deeper tumors. Others have reported correlation between BLI and tumor volume based on caliper measurements or MRI [11], [15], [23]. Recently, it was reported that high resolution ultrasound provided more accurate assessment of tumor volume than calipers, noting that skin thickness can be difficult to estimate [24].

In common with previous reports, ATO caused dose dependant vascular shutdown [6], [25]. We found that a dose of ATO at 5 mg/kg caused reduced vascular perfusion and the effect was increased at 7, 8, or 10 mg/kg (Figure 2), but the highest dose was often lethal within 24 hours. The response of PC3 and U87 tumors was quite similar, whereas MCF7 appeared more resistant with less effect at each time point (Figure 5). Extensive previous literature has shown efficacy on cultured cells of each of the lines examined here. Exposure ≥2 µM ATO reportedly induced cell death by apoptosis in MCF7 cells [26]. An IC_50_ = 2.5 µM was reported for PC3 cell growth and daily doses of 5 mg/kg IP significantly delayed growth of orthotopic PC3 tumors growing as xenografts in SCID mice [27]. Doses of 5 mg/kg IP daily given to mice with SC U87 gliomas caused a significant tumor growth delay when combined with irradiation (5×250 cGy) within 4 hrs, but failed to reach a significant effect when given alone (p<0.07) [28].

Anti vascular effects of ATO were extensively reported based on uptake of intravenously injected ^86^RbCl or clearance of ^99m^TcO_4_^−^ following direct intra tumor injection [6], [19]. A dose of 8 mg/kg yielded a 50% decrease in tumor blood perfusion at 2 hrs and 6 hrs in SCK tumors in mice with substantial recovery by 24 hrs, while FSAII tumors were somewhat more sensitive with 80% decrease at 2 hrs and only 40% recovery at 24 hrs [29]. A dose as low as 2 mg/kg caused 40% reduction of ^86^Rb uptake in FSaII tumors at 2 hrs [6]. However, ATO alone caused no tumor growth delay. Administration of ATO (8 mg/kg) 2 to 6 hrs before hyperthermia produced significantly enhanced tumor growth delay. ^99m^Tc clearance could be observed by sequential radio counting of tumors over time, whereas ^86^Rb uptake was based on sacrifice and analysis *ex vivo* [19].

Non-invasive assessment of vascular integrity is inherently more attractive and vascular disruption has been reported for several tumor types growing in diverse locations in various rodents. Using Laser Doppler flowmetry Hines-Peralta *et al* showed effects at doses as low as 1 mg/kg against VX2 tumors implanted intra renally in rabbits [25]. Orthotopic mammary R3230 tumors in rats and subcutaneous RCC tumors in mice required somewhat higher doses with blood flow reduced by about 40% following 3.5 mg/kg. Some of this difference may have resulted from the site of implantation. Several reports have used a dose of 8 mg/kg [5], [29], [30], though others have used 10 mg/kg [4]. Compared with the early studies based on uptake or clearance of radiolabels imaging requires far fewer tumor-bearing animals. More broadly Goertz *et al.* demonstrated the use of high frequency PD US to evaluate VDAs [14]. Power Doppler is technically more challenging than BLI, but has several potential advantages; notably, it avoids the need for luciferase expressing cells and is therefore applicable to primary human tumor xenografts, or indeed could be applied to human clinical trials. It also reveals 3D structure of vascular extent. Here, we applied PD US to the MCF7 tumors, which appear particularly well vascularized. In some tumor types vascular perfusion appeared too sluggish to effectively apply PD US even under baseline condition (*e.g.*, MTLn3 growing in rat), but infusion of contrast microbubbles did reveal vasculature and impairment following VDA administration, notably CA1P [2].

Dynamic BLI is particularly facile to implement and allows high throughput analysis. Three to five mice may be observed simultaneously although each imaging session does require fresh administration of luciferin. Several reports indicate that IV infusion provides higher signal, though it is quite transient and requires the technical skill of IV tail vein injection on multiple, successive occasions [13], [31], [32]. We favor subcutaneous administration in the foreback neck region, which gives highly reproducible BLI signals (Figure 3), as also reported previously [20]. Traditional BLI acquires images at a single time point post administration of luciferin, but acquiring the dynamic time course of signal evolution provides a more comprehensive perspective on vascular patency. We did note that the BLI signal had not reached the maximum value for the U87-luc-mCherry 7 tumor at 16 minutes (Figure 2). Thus, we extended the acquisition time for MCF7-luc-mCherry, although in this case the maximum was found at about 10 minutes (Figures 3, 4).

Dynamic BLI was previously used to characterize vascular disruption of MDA-MB-231-luc tumors following administration of CA4P and observations were validated by reference to dynamic contrast enhanced MRI and histology [16]. Here, comparative measurements were provided by PD US and confirmed using histology. In our previous work we had used a home-built BLI system [16], [20], which allowed observation of only one mouse at a time. The commercial system makes signal acquisition and analysis much easier and allows up to five mice to be assessed simultaneously. In terms of practical application we also note that the price of luciferin has fallen considerably and luciferase expressing tumor cell lines are becoming more readily available.

Vascular shutdown was confirmed by histology based on distribution of the perfusion marker Hoechst 33342. Two hours after ATO administration (8 mg/kg) histology showed reduced perfusion (Figure 6). Similarly reduced vascular perfusion was reported for TLT (transplantable mouse liver tumors) using Patent blue dye and tumor excision following 5 mg/kg ATO [33]. We observed further reduced perfusion after 4 hrs, but extensive return after 24 hrs (Figure 6) matching the observations *in vivo* (Figure 4, 5). Vascular impairment judged by BLI and PD US was found to be closely matched (Figure 4). Fluorescent imaging showed no response up to six hours, as expected since no vascular delivery was involved and the mCherry protein is reported to have a half life of about 24 hrs [34]. An alternative FLI approach is conceivable based on infusion of a vascular label; notably we have preliminary data indicating that so-called DyCE (dynamic contrast enhanced) FLI following infusion of indocyanine green (ICG) is sensitive to tumor perfusion and responsive to vascular disruption [35]. Since ICG is approved for use in patients such an approach could be clinically feasible for superficial tumors and may be worth exploration.

Arsenic trioxide was used as a model agent here based on the reports that is causes acute vascular shutdown in solid tumors [6], [19]. Moreover, ATO (TRISENOX®) is FDA approved in the United States for treatment of relapsed and refractory acute promyelocytic leukemia (APL) patients [36], [37] and there are ongoing clinical trials for solid tumors including liver, brain, lung and breast cancers (ClinicalTrials.gov). Dose limiting toxicity has been widely reported to limit potential use of ATO, but new targeted formulations have been presented [38], [39]. Evaluation of the efficacy of such materials could be facilitated by non-invasive imaging procedures. There is also active development of alternative vascular disrupting agents seeking to enhance efficacy, reduce toxicity, and logically combine with additional therapeutic modalities. Indeed, dynamic BLI was recently applied to the novel tubulin-destabilizing agents BPR0L075 and KGP265 demonstrating vascular disruption in human breast cancer xenografts [40], [41].

In this study the effect of ATO was demonstrated non-invasively using optical and ultrasound imaging. The strong correlation between BLI as a pre-clinical tool and PD ultrasound as a potential clinical tool suggests the potential for both assessment of pre-clinical development of VDAs and a specific biomarker to demonstrate efficacy in patients.

## Supporting Information

Figure S1 Variation and reproducibility of optical signals with respect to growth in U87-mCherry-Luc tumors. Upper graphs: Variation of (A) FLI and (B) BLI photon signal intensities for a group of six tumors over a period of 30 days following implantation (mean ± SE). Lower graphs: Variation of (C) FLI and (D) BLI photon signal intensities for a group of six U87-mCherry-Luc tumors following administration of saline IP. Fresh luciferin was administered at each time point (mean ± SE).
(TIF)

Figure S2 FLI and BLI during growth of MCF7-Luc-mCherry and PC3-luc tumors. Dependence of signal intensity on tumor volume measured using calipers for individual MCF7-mCherry-luc tumors by (A) FLI (R^2^>0.9) and (B) BLI (R^2^>0.9); C) Correlation of FLI and BLI for MCF7-mCherry-luc tumors (R^2^>0.88). (D) Dependence of signal intensity on tumor volume measured using calipers for PC3-luc tumors (R^2^>0.9).
(TIF)

Figure S3 Correlation between B-mode US images and caliper-measured tumor volume: repeat images for a single MCF7-Luc-mCherry tumor and wire mesh analyses below. Graph shows strong correlation between US and caliper-measured tumor volumes (R^2^>0.8).
(TIF)

Figure S4 Vascular disruption assessed by BLI in PC3-luc tumor. Left) Variation in bioluminescent signal intensity from tumor on sequential occasions before and 2 hrs after administration of ATO (8 mg/kg IP). Right) Representative images acquired 10 mins after administration of fresh luciferin on each occasion.
(TIF)

Table S1 Correlations between techniques.
(DOC)
