# Supplementary figures and images for: Comparison of Optical and Power Doppler Ultrasound Imaging for Non-Invasive Evaluation of Arsenic Trioxide as a Vascular Disrupting Agent in Tumors
Source: PLoS One. 2012 Sep 28;7(9):e46106. doi: 10.1371/journal.pone.0046106 (PMC3460997; doi:10.1371/journal.pone.0046106)

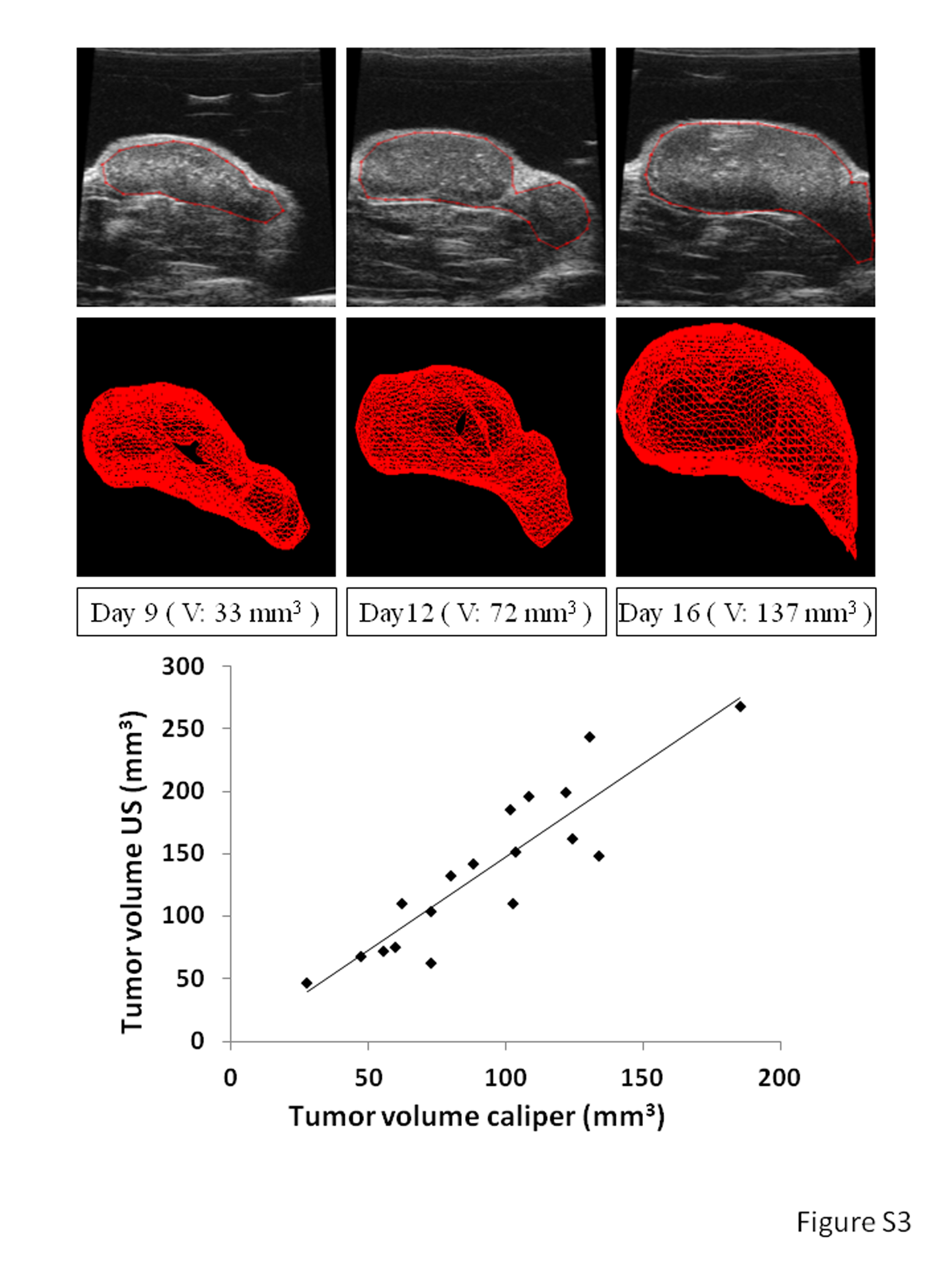

Supplement: Figure S3 — Correlation between B-mode US images and caliper-measured tumor volume: repeat images for a single MCF7-Luc-mCherry tumor and wire mesh analyses below. Graph shows strong correlation between US and caliper-measured tumor volumes (R2>0.8). (TIF) [file pone.0046106.s003.tif]

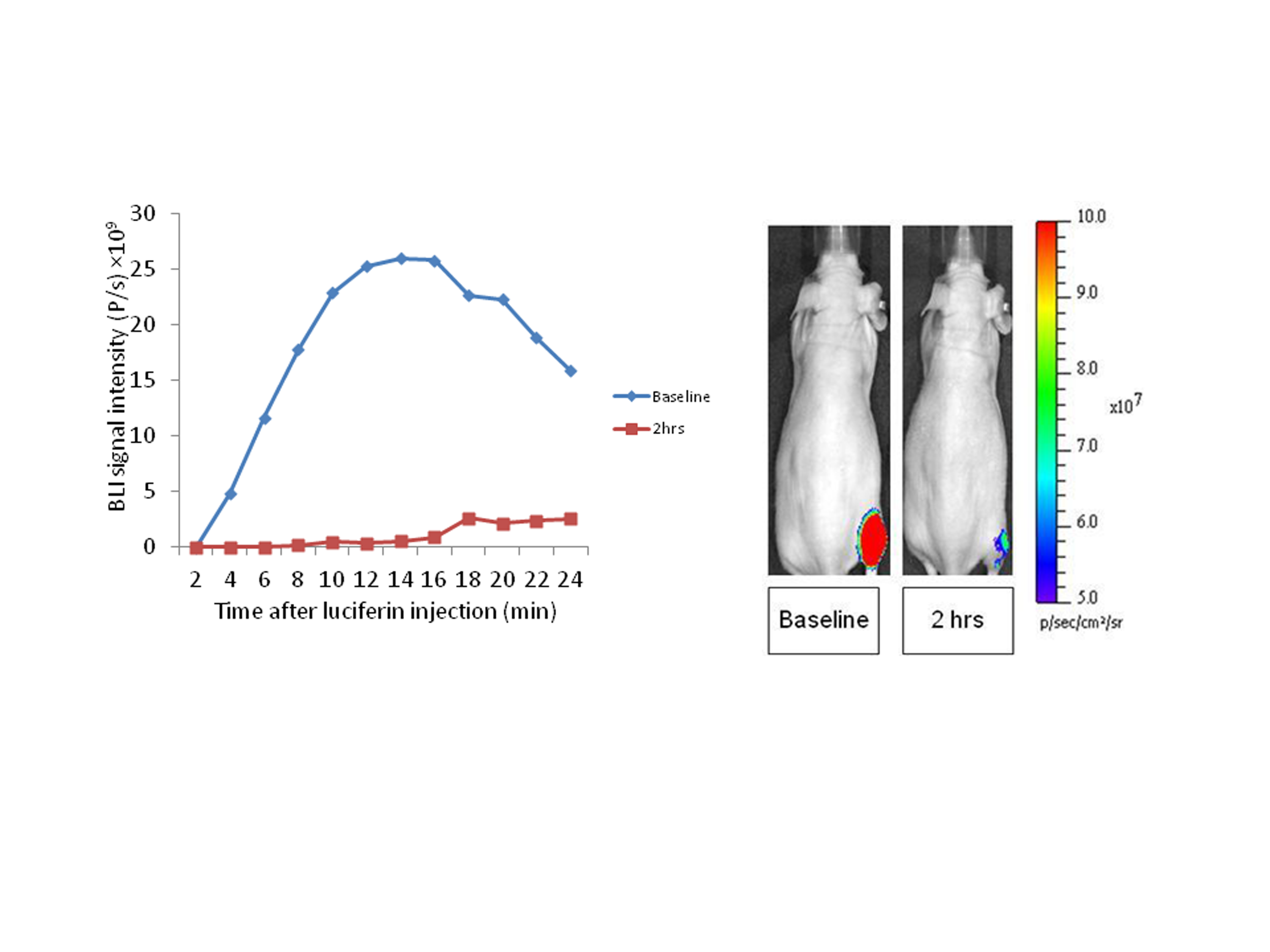

Supplement: Figure S4 — Vascular disruption assessed by BLI in PC3-luc tumor. Left) Variation in bioluminescent signal intensity from tumor on sequential occasions before and 2 hrs after administration of ATO (8 mg/kg IP). Right) Representative images acquired 10 mins after administration of fresh luciferin on each occasion. (TIF) [file pone.0046106.s004.tif]
