# Supplementary material for: Comparison of Optical and Power Doppler Ultrasound Imaging for Non-Invasive Evaluation of Arsenic Trioxide as a Vascular Disrupting Agent in Tumors
Source: PLoS One. 2012 Sep 28;7(9):e46106. doi: 10.1371/journal.pone.0046106 (PMC3460997; doi:10.1371/journal.pone.0046106)
Supplement: Table S1 — Correlations between techniques. (DOC) [file pone.0046106.s005.doc]

**Table S1 Correlations between Techniques**

| Techniques | R^2^ | P | Cell line | Number of subjects  (measurements over 30 days) |
| --- | --- | --- | --- | --- |
| FLI vs. caliper | >0.82 | <0.0026 | U87-Luc-mCherry | 6 (24) |
| BLI vs. caliper | >0.86 | <0.0012 | U87-Luc-mCherry | 6 (24) |
| FLI vs. BLI | >0.84 | <0.0034 | U87-Luc-mCherry | 6 (24) |
| FLI vs. caliper | >0.90 | <0.0228 | MCF7-luc-mCherry | 3 (6) |
| BLI vs. caliper | >0.90 | <0.0374 | MCF7-luc-mCherry | 3 (6) |
| FLI vs. BLI | >0.88 | <0.0118 | MCF7-luc-mCherry | 3 (6) |
| US volume vs. caliper | >0.80 | <0.0001 | MCF7-luc-mCherry | 6 (18) |
| BLI vs. PD | >0.78 | <0.1156 | MCF7-luc-mCherry/ PC3-Luc combined | 6 (6)  (4MCF7+2PC3) |
| BLI vs. caliper | >0.90 | <0.0079 | PC3-Luc | 3 (9) |
